# Transperineal excision of malignant peripheral nerve sheath tumors of the ischiorectal fossa: Case report of a rare tumor in a frequently forgotten anatomical region

**DOI:** 10.1016/j.ijscr.2023.108674

**Published:** 2023-08-18

**Authors:** Chiara Eberspacher, Stefano Arcieri, Enrico Coletta, Stefano Pontone, Francesco Leone Arcieri, Domenico Mascagni

**Affiliations:** Department of Surgery, University of Rome “Sapienza”, Rome, Italy

**Keywords:** Malignant peripheral nerve sheath tumors, Ischiorectal fossa tumor, Histological surprise, Case report

## Abstract

**Introduction and importance:**

Malignant peripheral nerve sheath tumor is an aggressive tumor that arises from peripheral nerves. Frequently associated with neurofibromatosis, its common localization is in the extremities, trunk (with paravertebral regions), neck and head. Some cases have been found in the pelvis or uterus. In this case report we illustrate one of the rarest localization of this type of tumor in the ischiorectal fossa, with the full recovery of the patient after surgical excision and radiotherapy.

**Case presentation:**

A 61-year-old woman showed a lump near the anus which was initially diagnosed as a lipoma of the right ischiorectal fossa, by Computed Tomography scan. The tumor was completely removed with a minimal skin incision, and the patient had a complete recovery. Only the pathological examination determined the diagnosis of malignant peripheral nerve sheath tumor, in this unusual localization. In consideration of its high aggressiveness the patient underwent radiotherapy. After more than two years of follow-up there is no sign of recurrence.

**Discussion:**

In sites far from branches of nerves, malignant peripheral nerve sheath tumors can be considered episodic. Ischiorectal fossa is a rare localization, and the differential diagnosis from benign mesenchymal cell tumors can be challenging. When possible, a biopsy should be performed before surgery.

**Conclusion:**

Surgical excision of tumors in ischiorectal fossa should be always complete, in consideration of possible histological surprise.

## Highlights

• Malignant peripheral nerve sheath tumors are very rare: this is the first description of localization in ischiorectal fossa.
• The histological diagnosis of malignant peripheral nerve sheath tumors can be a surprise.
• Malignant peripheral nerve sheath tumor of the ischiorectal fossa can be successfully excised with transperineal approach.
• Radiotherapy should be a completion of the therapy, even if the literature lacks studies in this sense.

## 1 Introduction

Malignant peripheral nerve sheath tumors (MPNSTs) develop from the branching of peripheral nerves, in particular from nerve sheath components (perineural cells, Schwann cells fibroblasts) [1]. They are both aggressive and rare: the sixth most common type of soft tissue sarcomas, with an incidence of 1 per 100,000 [2,3]. There is no gender difference in terms of prevalence, and the median age of onset is 35 years [4,5]. Approximately 50 % of these malignancies tend to arise sporadically, whereas the rest are associated with neurofibromatosis type 1 (NF1) or radiation exposure [6,7]. MPNSTs can arise in every site, but in the pelvic region are extremely rare [7] and no case of MPNSTs that originates in the ischiorectal region is described in the literature. In this report, we present an MPNST of the ischiorectal fossa, its treatment and follow-up. The patient was treated in our university polyclinic. This clinical case has been described according the SCARE criteria [8].

## 2 Presentation of the case

In November 2020, a 61-year-old female patient was submitted to an outpatient visit, at our university polyclinic, for a large perianal mass that had been increasing in size throughout the year. Her surgical history included cholecystectomy, thyroid nodulectomy for a benign nodule, appendectomy and tonsillectomy. Her medical history was otherwise unremarkable. There was not any case of NF1 in her family history. During a medical assessment, the patient complained of dull pain and discomfort in the buttock region, which was not associated with any defecation disorder. A careful clinical examination revealed a firm, tender mass with distinct boundaries. It extended from the right perianal region, close to the rectal wall, to the right gluteal region. The skin upon the lump did not show signs of inflammation. A digital rectal examination showed that the rectal mucosa was smooth but with an extraparietal palpable lump. There were no signs of internal bleeding. Anoscopy was negative for any macroscopic alterations. The results of the routine laboratory tests were normal.

**Fig. 1:**
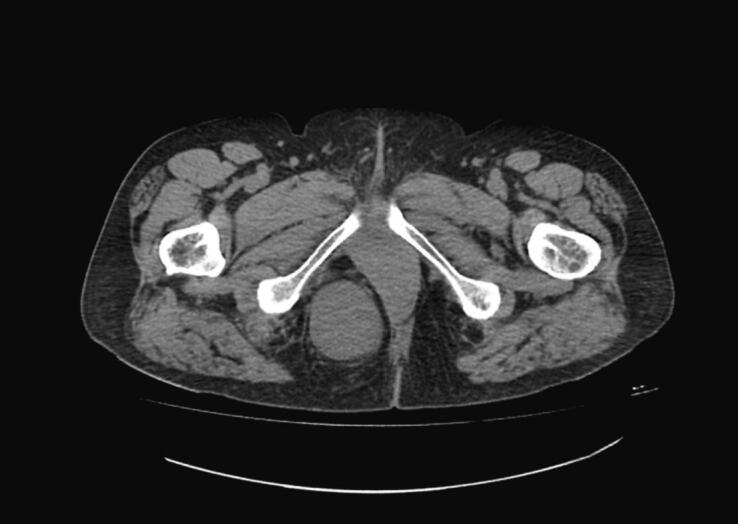
CT shows an 8,5 × 5,5 × 10 cm bilobed mass in the right ischiorectal fossa, extending into the right gluteal region.

**Fig. 2:**
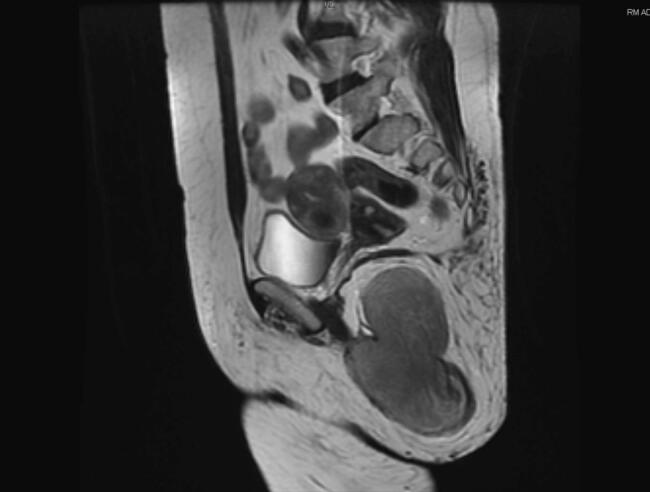
MRI shows a lump in the right ischiorectal fossa after contrast enhancement.

Contrast-enhanced Computed Tomography (CT) revealed, in the right ischiorectal fossa, a 8,5 × 5,5 × 10 cm bilobed mass, extending up to the gluteal region (Fig. 1). The mass displaced the uterus to the right, with no signs of bladder displacement. Enlarged pelvic lymph nodes, smaller than 10 mm, were identified. The patient's liver, spleen, pancreas and kidneys showed no abnormalities. Contrast-enhanced magnetic resonance imaging (MRI) of the lower abdomen showed the same finds of the CT scan. The final radiological diagnosis was a lipoma of the right ischiorectal fossa (Fig. 2).

**Fig. 3:**
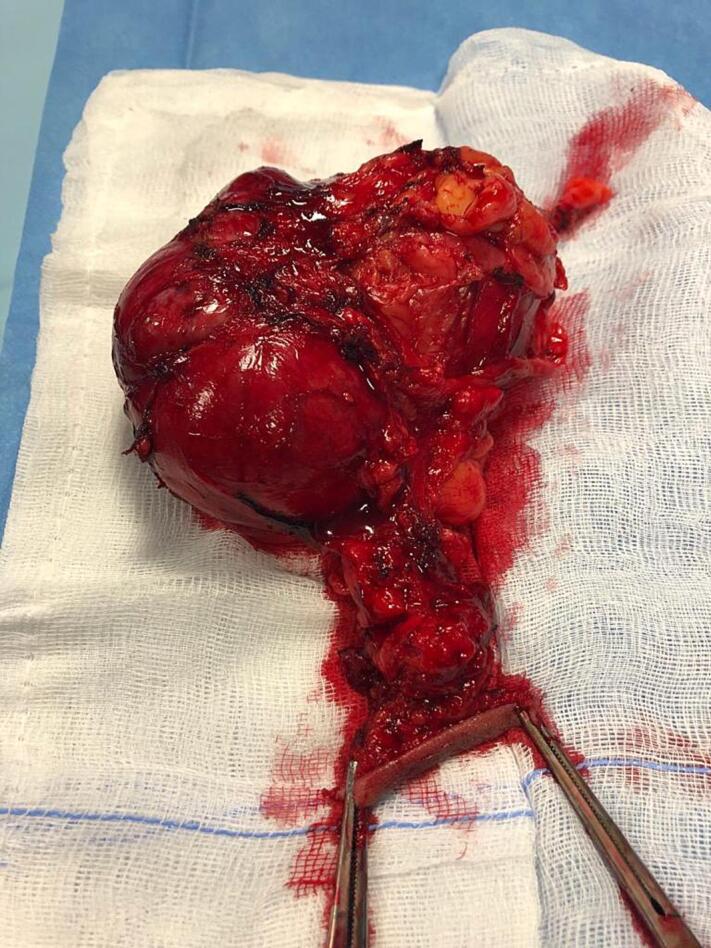
The pelvic mass appeared oval in shape and partially encapsulated, without any signs of necrosis.

The patient underwent a pelvic mass excision under a subarachnoid block. Intravenous prophylactic fluoroquinolones were administered during the induction. The patient's position was lithotomic: a right perianal 5 cm oval incision was made, including a portion of the skin overlying the mass. Intraoperatively, a firm, solid encapsulated mass of 9,5 × 7,5 × 5 cm was in the right ischiorectal fossa: the mass appeared from about 3 cm under the skin for an extension of about 10 cm. The tumor was entirely and accurately removed with an en-bloc excision (Fig. 3). The depth of the residual cavity in the ischiorectal fossa was of about 12 cm. The removal of the mass seemed complete as there was no element suggestive of infiltration at the level of the wall of the residual cavity and as the mass was clearly encapsulated. The specimen was histopathologically examined. The surgical procedure lasted 70 min and did not lead to any significant blood loss. A urinary catheter and drain in the right ischiorectal fossa were positioned.

Painkillers and antibiotics were administered in the postsurgical period. On the second postoperative day (PO), the urinary catheter was removed and the patient began an oral diet. On the fourth PO day the drain was removed, before the patient's discharge on the fifth PO day.

A macroscopic examination demonstrated an oval-shaped, partially encapsulated tumor with a stiff texture and no signs of necrosis. In the histopathology sections, with rich cellularity, spindle-shaped tumor cells were evident, with elongated nuclei, clear cytoplasm and moderate degrees of pleomorphism and atypia. The tissue appeared highly vascularized, with high levels of blood vessel tumor infiltration. The mitotic activity level was low with approximately three mitoses per 10 high-power fields. Microscopic necrotic areas were also found. Results of immunohistochemical staining showed positivity for vimentin, S-100 protein and CD-99. Negativity was observed for desmin, CD-34, CD-117, STAT-6, DOG-1, ALK, MDM-2 and smooth muscle actin. The Ki67 labeling index was <2 %. Resection of the tumor was complete, but the tumor was <1 mm from one of resected margins. The microscopic and immunohistochemical findings supported a final diagnosis of MPNST.

**Fig. 4:**
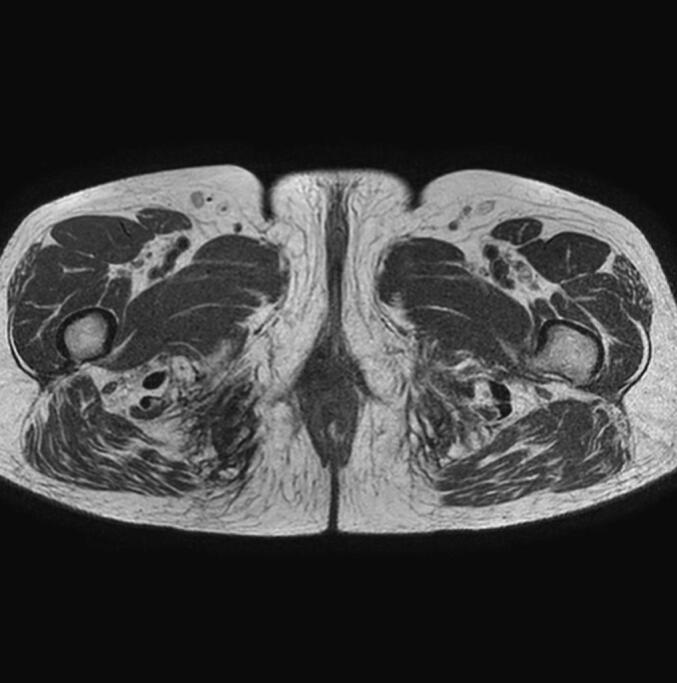
Complete surgical healing was achieved after the last MRI control.

With this surprising diagnosis and considering the high aggressiveness of this type of tumor, the patient underwent brachytherapy. After more than two years of follow-ups, the CT and MRI controls showed a complete resolution of MPNST, with no signs of recurrence (Fig. 4). Chronic limb pain after radiotherapy was the only residual symptom after 2 years of follow-up.

## 3 Discussion

MPNSTs in adults are associated with syndromes such as NF1, and these tumors are among the most fatal, arising between thirty and forty years of age [9]. Sporadic forms are rarer and arise usually around the age of 50; the majority of patients show an enlarged mass that may or may not be associated with neurological symptoms [10]. The purpose of surgery is total macroscopic excision, with tumor-free margins, and adjuvant radiotherapy can improve local tumor control [11].

MPNSTs are often episodic in different localizations. The most frequent localizations are along the major nerve roots of the trunk, extremities, head and neck [7,12]. Differential diagnoses of benign and malignant mesenchymal tumors can be challenging, as they appear to be nonspecific soft-tissue masses in imaging results [10]. Localization around the pelvis is relatively uncommon and is often reported in clinical case reports. A diagnosis is usually made only after surgical excision and a histopathological exam, without previous suspicion of an MPNST [13,14]. Localization in pelvic organs, such as the cervix, has been reported in some cases, with hysterectomy as the surgical treatment option [15].

Only a few case series of MPNSTs in the pelvis have been reported, and they focused on the differential diagnoses of benign tumors. For instance, Ogose et al. described 30 cases of peripheral nerve sheath tumors (both benign and malignant) in the pelvis [6]. They concluded that for the rarity of MPNSTs in this anatomical region, when anamnesis is negative for neurofibromatosis, preoperative diagnosis through imaging or fine needle aspiration can be very difficult. In their review of ischiorectal fossa tumors, Faria et al. mentioned that MPNSTs are among the rarest forms of tumors that arise from the nerves of the fossa; the authors also reported that the differentiation of MPNSTs from other malignant tumors of the fossa, such as liposarcoma, can be challenging, but they did not discuss any specific case [16]. According to some studies, percutaneous biopsy should be performed, when possible, to decide the correct therapeutic pathway [17].

The treatment of MPNSTs, especially those with unusual localizations, can be uncertain due to the lack of homogenous data [18]. The mortality rate is almost 43 % at 10 years, and survival is strongly linked to the primary or recurrent nature of the disease, tumor size and tumor site [4,19]. While clear margins are fundamental to reducing recurrence, the role of radiotherapy needs further study, especially in relation to local side effects [19].

In the case of our patient, the diagnosis was a surprise for various reasons: the patient did not report any signs of neurofibromatosis or other such syndrome. She did not report any important symptoms, excluding the presence of the mass at the perirectal level, and complained of mild dull pain, whereas one of the differential criteria for an MPNST is the presence of persistent pain or a neurological disorder [10]. Both CT and MRI indicated that the mass was a lipoma, with clear margins and cleavage surfaces from nearby organs (rectum, vagina and uterus); for this reason, we did not perform a core biopsy (in order to have a more significant sample of tissue), to investigate the histological type of the mass. The possibility of performing a perianal incision with the removal of the mass was excluded by two surgeons before our visit; nevertheless, a radical excision was realized during the operation. Due to the rarity and unique localization of MPNSTs, the histopathological response in this case was quite surprising to the surgical team, but the surgical outcome was achieved with adjuvant radiotherapy.

## 4 Conclusion

The ischiorectal fossa is a frequently neglected anatomical region, but a great variety of tumors can arise from its different components. MPNSTs are rare and difficult to diagnose, even when all correct imaging or preoperative biopsies are performed. In the present case, these two rarities were combined in the initial description of the MPNST of the ischiorectal fossa. Our approach to surgery, respecting surgical margins and the minimization of collateral damage, ensures that even when everything pointed to a benign pathology, the consequences of MPNST of the ischiorectal fossa, which was a histological surprise, could be addressed.

## Abbreviations

MPNSTs: Malignant peripheral nerve sheath tumors
NF1: neurofibromatosis type 1
CT: computed Tomography
MRI: Magnetic resonance imaging
PO: postoperative

## Ethical approval

IRB (Comitato Etico Territoriale LAZIO AREA 1) approved the article (Prot. 547/2023).

## Funding

There are no sources of funding to declare.

## Author contribution

Chiara Eberspacher: wrote the article, draft manuscript preparation, make important contribution in revision.

Stefano Arcieri: contributed to writing, gave final approbation.

Enrico Coletta, Francesco Leone Arcieri: contributed to the collection of the data, to the preparation of figures and the revision of literature; gave final approbation.

Stefano Pontone contributed with the revision of the literature in the discussion, the description of the clinical report gave final approbation.

Domenico Mascagni: designed the article in conception and design, wrote the article, gave final approbation.

## Guarantor

Chiara Eberspacher

## Research registration number UIN

Researchregistry8759.

## Availability of data and material

All original data and materials are available on request.

## Patient consent

Written informed consent was obtained from the patient for publication and any accompanying images. A copy of the written consent is available for review by the Editor-in-Chief of this journal on request.

## Conflict of interest

The authors declare that there is no conflict of interest.
